# Cognitive Dysfunction in Major Depressive Disorder. A Translational Review in Animal Models of the Disease

**DOI:** 10.3390/ph9010009

**Published:** 2016-02-17

**Authors:** Flavie Darcet, Alain M. Gardier, Raphael Gaillard, Denis J. David, Jean-Philippe Guilloux

**Affiliations:** 1 Université Paris-Saclay, University Paris-Sud, Faculté de Pharmacie, CESP, INSERM UMRS1178, Chatenay-Malabry 92296, France; flavie.darcet@u-psud.fr (F.D.); alain.gardier@u-psud.fr (A.M.G.); jean-philippe.guilloux@u-psud.fr (J.-P.G.); 2 Laboratoire de “Physiopathologie des maladies Psychiatriques”, Centre de Psychiatrie et Neurosciences U894, INSERM, Université Paris Descartes, Sorbonne Paris Cité, Paris 75014, France; R.GAILLARD@ch-sainte-anne.fr; 3 Service de Psychiatrie, Centre Hospitalier Sainte-Anne, Faculté de Médecine Paris Descartes, Université Paris Descartes, Sorbonne Paris Cité, Paris 75014, France; 4 Human Histopathology and Animal Models, Infection and Epidemiology Department, Institut Pasteur, Paris 75015, France

**Keywords:** major depressive disorder, cognitive dysfunctions, animal models of anxiety/depression, neurogenesis

## Abstract

Major Depressive Disorder (MDD) is the most common psychiatric disease, affecting millions of people worldwide. In addition to the well-defined depressive symptoms, patients suffering from MDD consistently complain about cognitive disturbances, significantly exacerbating the burden of this illness. Among cognitive symptoms, impairments in attention, working memory, learning and memory or executive functions are often reported. However, available data about the heterogeneity of MDD patients and magnitude of cognitive symptoms through the different phases of MDD remain difficult to summarize. Thus, the first part of this review briefly overviewed clinical studies, focusing on the cognitive dysfunctions depending on the MDD type. As animal models are essential translational tools for underpinning the mechanisms of cognitive deficits in MDD, the second part of this review synthetized preclinical studies observing cognitive deficits in different rodent models of anxiety/depression. For each cognitive domain, we determined whether deficits could be shared across models. Particularly, we established whether specific stress-related procedures or unspecific criteria (such as species, sex or age) could segregate common cognitive alteration across models. Finally, the role of adult hippocampal neurogenesis in rodents in cognitive dysfunctions during MDD state was also discussed.

## 1. Introduction

Cognitive dysfunction is a common feature of major depressive disorder (MDD), contributing to the serious decline in patients’ quality of life. Described in the 5th edition of the Diagnostic and Statistical Manual of Mental Disorders (DSM-V, [1]) as “significantly affecting the individual’s capacity to function”, these cognitive changes are associated with the set of emotional and behavioral alterations (including persistent depressed mood and loss of pleasure) that characterizes MDD pathology. Many clinical studies have focused their work on the nature and the magnitude of cognitive alterations during the clinical course of MDD [2,3,4,5,6]. During clinical observation, patients mostly report their difficulty to concentrate, to make decisions, the feeling that their brain is slowed down or the fact that “they forget everything” (tasks, meetings, *etc*.) [7]. These subjective complaints relate to a broad range of cognitive impairments reported during depressive episodes, from executive functions (attention, processing speed, cognitive flexibility) to working and visual learning and memory. However, contradictory findings from neuropsychological tests have encouraged clinicians to examine whether the heterogeneity of the MDD population would prevent from identifying a specific neurocognitive profile in depressed individuals.

The DSM-V clearly defines subtypes in major depression such as melancholic and atypical subtypes of MDD. Comparing different cognitive functions across MDD subtypes may help in the identification of neurocognitive patterns according to the specificity of MDD markers. Additionally, few studies aimed at delineating between trait- and state-like cognitive alterations, *i.e.*, the deficits observed exclusively during depressed episodes and those occurring prior, between and after MDD episodes [7]. One meta-analysis performed across first-episode MDD subjects, and including 13 studies, segregated state-dependent cognitive alterations in MDD subjects (psychomotor speed and memory functioning) from trait-markers (attention and executive functioning). However, the large clinical heterogeneity of MDD subjects may dampen this report.

In order for basic research to provide potential advances in this field, it is essential to use animal models that present behavioral, neurochemical and brain morphological phenotype reminiscent of some symptoms of MDD. Indeed, animal models exhibiting deficits in one or more of the relevant domains of cognition are useful to investigate mechanisms underlying impaired cognitive processes observed in MDD and their dependence on mood pathology. In rodents, many anxiety-depression models, including chronic early-life stress and adulthood models, have been validated for the study of anhedonic behaviors, modeling the negative mood symptomatology of MDD. It has been reported that adverse experiences during pregnancy or early stress life events in childhood could lead to an increased sensitivity to the effects of stress in adulthood life, directly enhancing vulnerability to depression [8,9]. Rodent models such as prenatal stress (chronic stress exposure during gestational period), early post-natal handling and maternal separation (chronic pups-dams separation during weaning period), have been validated to produce depression-like behavior in adulthood [10,11]. However, in most clinical cases, the apparition of MDD pathology occurs during adulthood and is typically caused by a succession of adverse stress episodes in life, leading progressively to the core of the pathology. Several adult animal models have been validated as anxiety-depression models such as social defeat model (SD) [12], learned helplessness (LH) [13], unpredictable chronic mild stress (UCMS) [14] and chronic corticosterone administration (CORT) [15]. Among these models, UCMS procedure, based on a chronic exposure of unpredictable stressors, has been reported as one of the most robust animal models of depression thanks to good predictive, face and construct validities [16,17].

In this review, the first part will be focus on clinical cognitive dysfunctions depending on the type and stage of the MDD illness. The second part of this work will gather preclinical studies observing cognitive deficits in different rodent models of anxiety/depression. For each cognitive domain, we will highlight which deficits can be shared, across models, and, whether specific stress-related procedures or non-specific criteria (species, sex, type of cognitive parameter measured) can segregate common cognitive alteration. Finally, the role of adult hippocampal neurogenesis in rodents in cognitive dysfunctions during MDD state will be discussed.

## 2. Cognition in Patients Suffering from MDD

### 2.1. Cognitive Performances in MDD through Different Ages

Cognitive abnormalities in many various cognitive domains have been reported among patients suffering from MDD [4,18,19,20,21,22]. Specifically, cognitive alterations in attentional processes [23,24,25], executive functioning [6,26,27,28], working memory [27,29,30], verbal or visual learning and memory [29,31] and emotional processing [31,32] were noted in MDD patients. While early-onset depression is associated with higher disease severity and with higher levels of recurrence [33], limited data are available regarding children, adolescents or young adults cognitive performances during MDD episodes. Deficit in attention, memory and problem solving could have a serious impact in these populations on daily activities, especially when individuals are involved in education or academics programs, during which their achievement depends on these skills [34]. Among the few studies examining cognitive performances in pediatric, adolescent or young adults depressed subjects, all of them agreed on a general cognitive degradation but none managed to extract specific impairments related to the early-onset of depression [33,35,36]. It remains currently unclear whether or not cognitive impairments should be considered as vulnerability markers of depression, potentially preceding the development of depressive symptoms or, whether cognitive symptoms develop only after the onset of a major depressive episode [33].

Further studies involving larger, homogenous cohorts of patients are needed to provide new understanding regarding this issue. Cognitive dysfunctions in elderly depressed people have been widely investigated, but no specific cognitive impairments have been observed due to the physiological decline of cognitive process with age and to potential neurodegenerative disorders appearing in late-life. However, late-life depression has been particularly associated with a slower speed in information processing, executive functions difficulties and working memory deficits [37,38,39]. Alternative treatments strategies including cognitive training, psychotherapy, assistive devices, interventional procedures, physical/speed therapy and others emerging therapies are employed to treat cognitive dysfunctions in elderly depressed patients, in addition to a classical pharmacological therapy [40].

### 2.2. Cognitive Neuropsychological Assessments Instruments Used for MDD Patients

The Hamilton Depression Rating Scale (HAM-D) and the Montgomery-Asberg Depression Rating Scale (MADRS) are clinician-administered assessments of depressive symptoms that are the most frequently used methods in depression clinical trials. However, neither of these scales evaluates cognition in any depth and both rely on a clinician’s subjective opinion based upon a patient’s report. In the HAM-D, a single item assesses psychomotor functioning, whereas a single item on the 10-item MADRS assesses concentration [41].

The number of studies investigating cognition performances in depressive disorder has grown during the last decade, reflecting the interest in cognition as a therapeutic target [42]. Given the extent and the magnitude of cognitive dysfunctions in MDD, a greater assessment of cognitive performances may help in MDD evaluation. However, little is known about current clinical routine practice and specific available assessment tools to assess cognitive symptoms in a depression context. A recent cross-sectional survey interrogating psychiatrists from different countries investigated the strategy and routine methods facing cognitive evaluation in MDD patients [43]. When psychiatrists were asked to share their assessment method to explore cognitive function in MDD, 61% of them exclusively relied on patient history interview, 32% of them used solely cognitive instruments and only 7% of them used both methods. Most of the psychiatrists who reported using instruments specifically cited the Mini Mental Status Examination (MMSE, preferentially used in dementia disorder such as Alzheimer’s disease) or instruments assessing depression severity rather than cognitive assessments tools (HAM-D, MADRS, Beck Depression Inventory or Geriatric Depression Scale). Only six appropriate cognitive assessment tools were mentioned, including the Trail Making Test, the Stroop test or the Digit span task. While this study showed psychiatrists’ awareness of cognitive dysfunction in MDD patients, few were actually using appropriate instruments and most of instruments cited were inappropriate for the intended population and disease state, evoking a general misuse and confusion regarding instruments for assessing cognitive dysfunction in MDD.

Through a review highlighting the nature of cognitive assessment instruments used in MDD trial, the California Verbal Learning task (CVLT), the Trail Making Test (TMT-A), the Wechsler Memory-Scale (WMS) and the Wechsler Adult Intelligence Scale (WAIS) were listed as the most frequently used in clinical studies [44]. Among cognitive existing batteries that assess several cognitive domains rather that one domain represented by a single task, the CANTAB battery (Cambridge Neuropsychological Test Automated Battery) is the most widely used in MDD trials.

Classified by cognitive domains, some of the most familiar used tests in the MDD context are as follows [41,45]:

- Attention processing monitoring: the Digit Span test and the Continuous Performance test,
- Processing speed: the Trail Making Test-Part A, the Digit symbol test and the Finger Tapping test,
- Executive functions and verbal memory: the Stroop Color Word test, the Trail Making Test-Part B and the Wisconsin Card Sorting Test,
- Memory functions: the Rey Auditory Verbal Learning Test, the Wechsler Memory Scale and the California Verbal Learning task

Despite the variety of accessible methods, there is still a lack of harmony regarding the practical use of appropriate instruments or batteries to assess cognitive functions in major depression. The development of a validated and standardized cognitive battery to use in a specific manner in MDD clinical trials is necessary to improve both assessments and treatments of MDD patients.

### 2.3. Progression of Cognitive Symptoms along the Course of MMD

**Figure 1:**
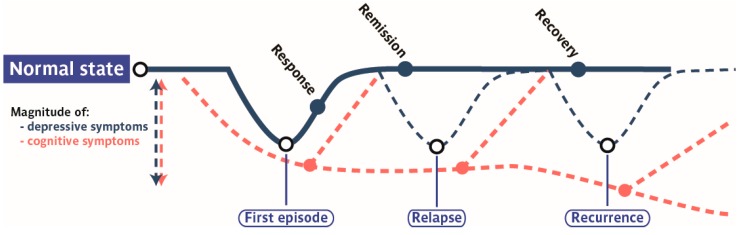
Schematic representation of a common trajectory towards chronic recurrent depression. Blue full and dotted lines represent various forms of progressions of depressive symptoms during the course of MDD. The red dotted line represents the magnitude of cognitive symptoms associated to MDD pathology according to MDD state. An early onset of cognitive symptoms depression has been reported before the clinical diagnosis. Those cognitive signs can persist even after remission or recovery of MDD symptoms.

The course of MDD pathology is characterized by a first-episode of major depression either followed by a complete remission or break periods without any mood-related symptoms and recurrent episodes separated by short time periods between relapse. In order to better visualize the progression of cognitive symptoms associated with depressive symptoms, Figure 1 simultaneously describes the course of depressive symptoms and the potential course of cognitive symptoms through different steps of the illness.

Although many studies found that a wide range of antidepressant treatments were able to improve both mood and cognitive-related symptoms in MDD [46,47,48], others failed to find an association between antidepressant treatment and improvement in cognitive functions in depressed patients [49,50,51]. Interestingly, the speed of remission of MDD seems related to an improvement in the patients’ cognitive functioning after a successful treatment. Indeed, unlike slow-remitters, the subjects with a rapid remission pattern presented specific cognitive benefits, with significant improvements in speed of information processing, working memory, and executive functions [52].

Although description of cognitive alterations in the context of mood disorders has been extensively analyzed in the literature [6,18,19,24,28,31,53], understanding why some cognitive dysfunctions are still present in remitted or recurrent state of MDD and how specifically progress these deficits remains unanswered. Indeed, recent reviews, focused on cognitive functioning in the remitted state of depression, confirmed that cognitive deficits persist in depressed patients despite the stabilization of mood-related symptoms [5,54]. These deficits seem to reside more within the cognitive domains of attention and executive functions (mental flexibility, decision-speed) than within other domains, although studies in additional patient cohorts are needed to strengthen this statement. The involvement of many factors associated to the progression of MDD pathology (number, duration and severity of MDD episodes) and other clinical features (age of onset, time elapsed since the last episode of depression, treatment interventions, sex or co-morbid psychiatric disorders) make the identification of a specific neurocognitive profile in remitted patients even more complex [5].

Although many studies found that a wide range of antidepressant treatments were able to improve both mood and cognitive-related symptoms in MDD [46,47,48], others failed to found an association between antidepressant treatment and improvement in cognitive functions in depressed patients [49,50,51].

The proportion of MDD patients that encountered recurrence episodes is estimated at 50%–60% [55]. Cognitive symptoms in recurrent depressive disorder (rDD) represent the largest range of cognitive impairments during the MDD pathology with decrements in memory, learning, attention, spatial visualization, visual-motor coordination, verbal fluency and memory, psychomotor retardation and most of the executive functions (planning, problem solving, behavioral inhibition, mental flexibility) [56,57,58]. A way to examine the degree of cognitive deficits in recurrent depressive disorders is to gather studies that compare first-MDD episode cognitive symptoms to recurrent depressive cognitive symptoms. A recent study evaluated the following cognitive functions: information processing speed (Digit Symbol from WAIS-R), executive functions and working memory (TMT, Stroop test), verbal memory (immediate and delayed memory) and learning ability (CVLT), and verbal fluency (VFT) in first-MDD episode and in recurrent episode patients [58]. Although no difference was found in the severity of the depressive symptoms between first-episode and rDD individuals, the patients from the latter group recorded significantly lower scores in all the cognitive tests. These findings are in line with a previous report, in which recurrent patients exhibited worse performances in all the tests (executive functions and perseverative tendency) compared to controls subjects [59]. Moreover, the number of recurrent episodes seems correlated with the severity of perseverative behavior. The cumulative duration of depressive episodes and their repetition have a detrimental effect on the severity of the associated-cognitive deficits.

Otherwise, evidence suggesting that rDD was associated with negative biases and increased sensitivity to negative events. While first-episode MDD patients can show an impairment in emotional cognitive function [31,32], rDD subjects displayed a deficit in identifying facial expression at a more severe degree, even demonstrating excitation and exaggeration for sad emotions [60]. Thus, neural mechanisms involved in the perception of negative or happy/neutral faces may differ, confirming the abnormal neural processing of emotional stimuli in depressed patients all along the course of the disease. 

Taken together, rDD are characterized by a proportional aggravation of cognitive disturbances compared to first-episode MDD (Figure 1). This progressive decline of cognitive functioning through MDD illness may contribute to the failure to reach a full recovery in patients and considerably widens their risks of developing over time dementia and neurodegenerative disorders.

Many questions about cognitive deficits associated with MDD pathology remain unanswered: could cognitive impairments be a premature behavioral marker of susceptibility to depression and be used as a prevention tool? Are residual cognitive dysfunctions in remitted patients a risk factor for relapse? New discoveries in this field could significantly improve therapeutic care in MDD patients. Relieving cognitive dysfunctions may be a necessarily step to treat major depression.

### 2.4. Cognitive Dysfunctions According to the MDD Subtypes

Most of clinical studies investigating cognitive dysfunctions in MDD patients did not detail the heterogeneous condition of depression symptomatology. Melancholia and atypical disorders are the main subtypes in depression. Specifically, melancholic depression can be distinguish from non-melancholia by a set of physical symptoms occurring during clinical depressive episodes such as anhedonia and/or lack of reactivity to usually pleasurable stimuli, observable psychomotor retardation, weight loss, worsening of symptoms in the morning hours and early morning awaking (DSM-V, [1,61,62]). On the other hand, atypical subtype is rather characterized by mood reactivity and positivity, a younger onset of age, less severe and fewer depressive episodes, longer duration of episodes, hypersomnia, weight gain and more co-morbidity with anxiety and substance abuse [1,63]. In addition to the DSM-V definition, distinct biological correlates differentiate melancholic subtype from others subtypes of depression. Melancholic depression is associated with HPA-axis hyperactivity (hypercortisolemia) and specific sleep patterns (reduced REM latency, increased REM time and reduced deep sleep) [64,65].

After two decades of research, increasing evidence suggests that melancholic patients exhibit greater cognitive dysfunctions than non-melancholic patients but no qualitative review focusing on cognitive impairments in melancholic subtype has been written yet. Table 1 summarizes clinical studies evaluating cognitive functions within melancholic patients through various neuropsychological tests. Classical clinical assessments to identify melancholic patients in these studies were determined by DSM-IV or DSM-V criteria for melancholic features Checklist (DSM-IV) and the CORE index for melancholia by behavioral observation. The CORE index was developed in 2007 when observable psychomotor disturbance was judged as the most relevant clinical markers of melancholic depression and psychomotor retardation was announced as the “core” behavioral pattern defining melancholic MDD [61,66]. This assessment was preceded by a 17-item HAM-D evaluation and, occasionally, the Mini Mental State Examination (MMSE) was employed as a screening tool to exclude potential subjects with early onset dementia.

**Table 1:** Differential cognitive impairments in melancholic and non-melancholic patients.

MDD Type	Sex	Mean Age (Years)	Cognitive Domains Tested	Assessment Methods	Main Results	Reference
MEL (*n* = 20) Non-MEL (*n* = 18) CTRL (*n* = 38)	♂/♀	39	Psychomotor tasks	Fitt’s task Figure-copying task Symbol digit substitution task	MEL patients were slower performing all the tasks compared to non-MEL and CTRL patients.	[67]
MEL (*n* = 7) Non-MEL (*n* = 8) CTRL (*n* = 8)	♂/♀	40	Response selection Attention Executive function	Choice reaction task (T1) Spatial Stroop task SRC task Spatial Stroop + SRC task	- MEL patients were significantly slower than non-MEL patients in T2, T3, and T4.MEL patients were slower than CTRL in all tasksNo difference between non-MEL and CTRL in all tasks	[68]
MEL (*n* = 11-20) Non-MEL (*n* = 11–20) CTRL (*n* = 11–20)	♀	50	Executive function Memory	CANTAB battery: ID/ED set shifting task, SOC Spatial Recognition Memory, PAL	- MEL patients showed deficit in executive function in the ID/ED set shifting compared to non-MEL patients;No difference between groups in SOC, PAL, SRC	[69,70]
MEL (*n* = 26) Non-MEL (*n* = 9) CTRL (*n* = 26)	♀	34	Explicit episodic memory Implicit (procedural) learning	WMS-R SRTT	- No difference between MEL and non-MELSequence-specific implicit learning was lower in MEL compared to non-MEL	[71]
MEL (*n* = 17) Non-MEL (*n* = 17)	♂/♀	41	IQ Executive function Attention/working memory Learning/long-term verbal memory Prospective memory Attention, response inhibition Set shifting, feedback use Semantic memory/ verbal fluency Planning, self-monitoring, multi-tasking	NART Donders Simple Reaction Time Digit Span test CVLT Prospective memory task SCWT Shortened WCST COWAT SET	*Baseline MDD*: MEL patients showed deficits in memory tasks (CVLT trial 1, CVLT total trials) and prospective memory (delayed free recall). MEL group recalled fewer words overall; MEL patients performed more poorly than non-MEL group in executive functions tests: digit span backwards, SCWT interference score, WCST categories completed, WCST perseverative errors and modified Six Elements Test; *Remitted MDD*: Same cognitive impairments in MEL patients were observed.	[72]
MEL (*n* = 20) Non-MEL (*n* = 20) CTRL (*n* = 20)	♂/♀	47	Working memory Emotion classification Arousal and valence rating	Emotion face paradigms	MEL patients showed better memory for sad faces (sad benefit).	[73]
MEL (*n* = 65) Non-MEL (*n* = 59) CTRL (*n* = 124)	♂/♀	39	Memory recall Attention Short-term working memory Executive functioning Semantic knowledge, language Spatial visual memory Cognitive flexibility, selective attention	Verbal recall, recognition task Time estimation task Reverse digit span task Executive maze task Word Generation test Span of visual memory task Verbal Interference test Switching of attention task	MEL patients showed poorer performances in spatial visual memory and attention task compared to non-MEL group.	[74])
MEL (*n* = 142) Atypical (*n* = 76) Undifferentiated (*n* = 91) CTRL (*n* = 200)	♂/♀	34	Processing speed Attention Shifting Planning Verbal fluency Visual spatial memory Verbal working memory	TMT-A, Digit symbol coding subtest Digit Span Forward of the WAIS-RC Modified WCST, TMT-BTower of Hanoi (TOH) Animal naming WMR-RC Digit span backward subtest of the WAIS-RC	MDD state: In the domains of processing speed (TMT-A and Digital Symbol Coding of WAIS-RC) and verbal fluency (animal naming), MEL patients performed significantly worse than atypical patients; Remitted state : MEL patients performed worse than controls in processing speed, shifting and verbal fluency.	[63]
MEL (*n* = 25) Non-MEL (*n* = 63)	♂/♀	48	Attention Information processing speed/Mental flexibility Psychomotor speed Response inhibition Planning Working memory Semantic verbal fluency	TMT A-B; WAIS-ITMT A-B ; SCWT I/IIFinger Tapping test SCWT III Tower of London WAIS-I/WAIS-IINaming	Baseline: MEL patients performed worse than non-MEL patients in most of neuropsychological tests (TMT-B; WAIS I/II, SCWT, TOL and in the Finger Tapping Test); Remitted state: Overall, MEL patients performed worse than non-MEL patients. Significant differences were found in the TMT-B and the semantic verbal fluency.	[62]
MEL (*n* = 279) Non-MEL (*n* = 544) CTRL (*n* = 247)	♂/♀	37	Motor coordination Response inhibition Sustained attention Decision speed Information processing Verbal memory Working memory Executive function Cognitive flexibility Explicit emotion identification Implicit emotion identification	Finger tapping Go-NoGo Continuous performance task Choice reaction time task Switching attention Memory recall and cognition Digit span task Maze task Verbal interference Identification accuracy/RTPriming RT	MEL patients performed worse at switching attention, decision speed and verbal interference compared to non-MEL patients; MEL patients were significantly slower than non-MEL patients to identify happy faces in explicit and implicit emotion identification.	[75]

Abbreviations: MEL: melancholic; non-MEL: non-melancholic; CTRL: Controls; SCR: Stimulus-Response Compatibility; CANTAB: Cambridge Neuropsychological Test Automated Battery; SOC: stocking of Cambridge task; ID/ED: intradimensional/extradimensional attention set shifting task; SRM: spatial recognition memory; PAL: paired associated learning task; WMS-R: Wechsler Memory Scale-Revised; SRTT: Serial Reaction Time Task; NART: National Adult Reading test; CVLT: California Verbal Learning task; PM: Prospective Memory task; SCWT: Stroop Color Word Test; WCST: Wisconsin Card Sorting Test; COWAT: Controlled Oral Word Association Test; SET: Modified Six Elements Test; TMT (A or B): Trail Making Test; WAIS-RC: Wechsler Adult Intelligence Scale—Revised by China; TOH: Tower of Hanoi; WMR-RC: Wechsler Memory Scale-Revised by China; RT: reaction time.

Conducted in small cohorts of patients, the oldest studies mainly focused on psychomotor and motor coordination performances such as Finger tapping task, fast walk, finger-thumb apposition, hands movements, foot tapping, finger-copying task or reaction time tasks. In accordance to the CORE concept, psychomotor performances were diminished in melancholic patients compared to non-melancholic patients [67,68,76].

The major finding emerging from the variety of cognitive domains tested is that all studies assessing attentional processes and more globally executive functions invariably showed deficits in one or several associated tasks in melancholic patients compared to non-melancholic patients [62,63,68,70,71,72,74,75]. These specific impairments are already a key component in melancholic profile. It also appears that the differentiation between melancholic and non-melancholic groups relies on increased task difficulty. Indeed, simple reaction task [68], spatial recognition memory [69,70], explicit episodic memory [71], semantic memory [72], memory recall or working memory [74,75] were similar across depression subtypes. In contrast, complex tasks that required set-shifting [62,69,70], attentional processes [62,68,71,72,74], cognitive flexibility [72,75], planning [62], decision-speed [63,75] distinguish melancholic and non-melancholic MDD patients.

As mentioned previously, cognitive abnormalities in MDD patients can persist even after clinical remission [5,54,77,78]. So far, only two studies compared cognitive performances of MDD patients with or without melancholic features longitudinally, from admission to recovery [62,72]. At baseline, both investigations agreed that cognitive performances in melancholic were impaired in most of the cognitive domains tested (attentional processes, information processing, psychomotor speed, mental flexibility, learning verbal memory, planning) compared to non-melancholic subtype. Interestingly, these memory and executive dysfunctions in melancholic patients were maintained despite the remitted state of patients. In contrast, some of cognitive disturbances observed in non-melancholic patients were improved after remission [62,72]. The persistence of some cognitive dysfunctions, mostly reported in melancholic patients, could represent a marker of a distinct depressive subtype instead of being secondary to the severity of depression. Further investigations are needed to determine why melancholic patients require a longer period of recovery in order for their cognitive functions to be restored to control levels. Only one study investigated cognitive deficit in the three different MDD subtypes defined by DSM-V: melancholic, atypical and undifferentiated patients. Major depressive subtypes displayed similar cognitive deficits in most of the tested domains but differed in some cognitive tasks. Melancholic patients performed significantly worse in domains such as processing speed (TMT-A, Digit symbol coding subtest) and verbal fluency (animal naming) compared to atypical and undifferentiated patients [63]. After remission from depression, while all the subtypes could recover their visual spatial memory and verbal working memory, clinically remitted melancholic patients exhibited specific deficits in processing speed, set-shifting measures and verbal fluency, in line with previously discussed results [62,72], suggesting once again a distinct profile in neurocognitive performance in baseline and remitted MDD, depending on the subtype.

There is substantial evidence showing that perception in patients with MDD is characterized by blunted responsiveness to emotionally positive information as well as an increased tendency to perceive emotionally neutral visual information as negative [79]. However, relatively few studies have used neurobehavioral measures to examine emotional disturbances and loss of positive affect in melancholic MDD population. When melancholic subjects were asked to classify emotional faces previously encountered, they showed either a better memory for sad faces [73] or significantly more time at explicitly identifying happy faces [75] compared to non-melancholic patients. These findings support that melancholic patients display a hypersensitivity to sad stimuli, reflected in a greater tendency to recall or identify sad expressions. Perceptual emotion biases appear to be specific in melancholic patients and could open up the debate about a possible primary neurocognitive feature of melancholic depression instead of a consequence of symptomatic mood. Further longitudinal neuroimaging studies combined with cognitive measures in melancholic patients could provide new insights in this field. 

Taken together, the majority of studies agreed on both generalized and specific cognitive impairments in the melancholic subtype, characterized by a greater degree of these alterations compared to non-melancholic patients. Specifically, melancholic patients were distinguished by specific impairments in decision speed and positive emotion processing [63,73,74,75]. Moreover, given that most of cognitive deficits persist in remitted state in melancholic patients, the debate about the rehabilitation process of this type of depressive patients warrants an important focus.

Although subtyping depressive patients can be a useful tool in identifying specific profiles in depressive symptomatology, its clinical utility regarding predictive antidepressant drug efficacy must be hampered by recent exploratory [80] or translational research [81] investigations.

Even though, anhedonia and dysfunctional reward processing are unspecific to MDD, and may be observable in other psychiatric disorders [82], this is one of two required symptoms for a diagnosis of MDD [1]. This decrease in engagement in rewarding activities may be related to the complex disturbances in the emotional, motivational and cognitive domains that are associated with depression. Changes in reward-motivated learning in depression may exacerbate the clinical symptoms, or delay recovery. MDD and especially MDD-anhedonic subjects can display deficits in motivational processing and dysregulation of the brain's reward system [83]. Studies in MDD subjects showed a deficit in establishing a reward bias [84], and in reward-related decision-making [85]. If the assessment of “motivational anhedonia” is improved, it could help patient making behavioral choices that are likely to increase exposure to positively reinforcing experiences. Indeed, alterations in reward-related learning failed to appropriately guide or motivate subsequent behavior. These forms of incentive learning depend on several factors, including reward processing, motivation and the neuroplasticity underlying formation of new or strengthened memories. However, anhedonia is a particularly difficult symptom to treat, especially with current first-line pharmacotherapies (e.g., SSRIs), that do not adequately address motivational anhedonia in depression ([86] for review). If little is known about reward-motivated learning in animal models, a recent study using chronic exposure to corticosterone-induced depression-like behavior in Rat produced lasting deficits in the acquisition of reward-related learning tested on a food-motivated instrumental task [87]. Interestingly, in the same study, authors showed that amitriptyline increased instrumental performance compared in corticosterone-exposed rats. This may suggest that chronic exposure to amitriptyline *per se* facilitates this form of reward-related learning. The interrelationship between specific components of motivational dysfunction and specific cognitive systems in the context of depression remains to be studied in order to allow for a greater understanding of mood disorders [88].

## 3. Cognitive Behavioral Paradigms Used to Assess Learning and Memory Performances in Rodent Models of MDD

Cognition is a complex brain function that involves processes including attention, processing speed, learning and memory, working memory, verbal fluency and executive functions. However, some of these cognitive domains cannot be modeled in Rodents because of the higher cognitive demands specific to Humans, particularly in executive functions such as planning, problem solving, multi-tasking or decision-making and verbal fluency.

Collective efforts from preclinical researchers and clinicians are currently in progress to improve the translation of fundamental research tools into clinical application in Psychiatry. For example, the Cognitive Neuroscience Treatment Research to Improve Cognition in Schizophrenia (CNTRICS) initiative was introduced to identify tasks with construct validity across species to improve the “translational-ity” of behavioral tests from rodents to non-human primates and humans [89,90]. More recently, the Research Domain Criteria (RDoC) project, established by the National Institute of Mental Health, propose a biologically-valid framework for the understanding mental disorders. This latter project relies on functional dimension of behavior based on genes, molecules, cells, circuits, physiology, behavior and self-reports, like the “Cognitive Systems” domain, and will allow a better crosstalk between clinical and preclinical studies [81,91]. In preclinical research, the development of visual touchscreen-based memory procedures to assess cognitive functions in animal models will improve standardization of testing approaches, stimuli and conditions and will minimize experimenter involvement and potential bias. These types of behavioral tasks already exist to assess working memory in Rodents [92] but they are not used yet in animal models of anxiety/depression.

Despite this growing awareness for translational research across species, classical learning and memory behavioral paradigms remain widely used in fundamental research and in the context of anxiety-depression. Table 2 summarizes typical behavioral paradigms used in Rodents to assess cognition in anxiety/depression models of animals.

**Table 2:** Behavioral paradigms in rodents used to assess cognitive functions in anxiety/depression models.

Cognitive Domains and Functions	Behavioral Paradigms in Rodents
Attention	5-choice serial reaction time task (5-CSRTT)
Executive function - cognitive flexibility - inhibitory learning	Attentional set-shifting task (ASST) Reversal Morris water maze Prepulse inhibition (PPI)
Learning and memory	
Working memory	Delayed alternation Y-maze Delayed alternation T-maze Delayed match-to-sample with odors, subjects Modified MWM, BM, RAWM, RAM
Episodic memory	Novel object recognition test Object location recognition Passive avoidance place Social discrimination procedure
Reference spatial memory	Morris water maze (MWM) Barnes maze (BM) Radial arm water maze (RAWM) Object location recognition/ Object-in-place
Associative memory	Contextual/cued fear conditioning Extinction fear conditioning Passive/active avoidance place

Among them, few have a strong translational value from rat to non-human primates to humans (5-choice serial reaction time task to assess attentional process). In addition, fear extinction conditioning is the only task that presents a direct translational assessment from rodents to humans. Except these paradigms, the vast majority of preclinical assessment tools does not exhibit particular translational features. In exchange, they offer the advantage to be very well-validated through literature history, thus allowing relevant comparisons between studies using the same cognitive task.

Finally, some of the behavioral tests appear several times in different cognitive domains (*i.e.*, object location recognition, passive avoidance), confirming that a unique categorization of a cognitive task into a specific domain of memory is not always appropriate.

## 4. Episodic Memory in Rodent Models of Anxiety/Depression

In humans, episodic or autobiographical memory refers to the recollection of personally experienced past events and facts, which can be ranged from specific and general memories. Specific memories occur on a particular day at a specific place and time, whereas general memories recall events that occurred repeatedly or events that lasted more than a day. Overgeneral memory, characterized by subjects recalling fewer specific episodic details and prioritizing general schematic events has been observed in individuals with depression [93,94]. Thus, impairment in episodic autobiographical memory has become a consistent feature in MDD patients. In addition, overgeneral autobiographical memory was found to be a predictor of the course of depression in a meta-analysis [95] and to be persistent in remitted or recurrent phases of MDD [96,97]. Finally, a recent work found that children of mothers with a history of MDD during their child’s life, recalled less-specific memories, pointed out overgeneral memory as a cognitive vulnerability for depression [98]. However, the majority of neuropsychological tests used to evaluate cognitive functions in depressed patients do not include episodic memory assessment. 

Considering the conscious approach of episodic memory assessment in Humans, translational research in Rodents required an adaptation in terminology, finally using “episodic-like” memory to designate animal’s performances. Recognition memory tasks are widely used tools to assess episodic-like memory in rodents. These behavioral paradigms rely on animal’s ability to determine whether or not a stimulus has been previously encountered. The type of stimulus (nature of objects, location of objects, order of presentation) can vary to evaluate the various components of episodic-like memory (What? Where? When?). The spontaneous nature of this task, exploiting the curiosity of rodents and their propensity for novelty seeking, confers to recognition tests many advantages, especially in an anxiety-depression context. Unlike many others learning or memory paradigms, no positive (food reward) or negative (punishment, water) reinforcements are needed, minimizing other psychological parameters that could influence performances during the task [99]. However, modeling MDD conditions in rodents induces some modifications in parameters such as anxiety, motivation and motor activity [15,100,101] that cannot be excluded for the interpretation of the performance in recognition tasks. The involvement of these parameters is systematically corrected with a measure of motor activity either during the test itself (total ambulatory distance, number of crossings in the arena) or in other behavioral paradigms applied before memory assessment (rotarod, Open Field) [99,102]. When the anxious state overly restricts exploration behavior, adaptations in the protocol test must be set up to re-establish a sufficient amount of exploration. For example, introducing one or several habituation periods (animals exploring the arena empty without objects) preceding the acquisition and the retention period of the test can fix this issue [99].

### 4.1. WHAT?

Many preclinical studies have investigated the performances of anxiety-depression models on object recognition memory (Table 3). The novel object recognition test (NORT), initially described by Ennaceur and Delacour [103] usually comprises an acquisition session with two identical objects and a retention session during which animals have to discriminate between a familiar object previously faced and a recently introduced new object (different in size, shape, texture, colors). Overall, findings differ strongly across and inside animal models [104]. Antenatal and early post-natal chronic stress procedures (PNS, MS, MD) present the most conflicting findings when compared to chronic adult stress procedure (SD, UCMS and CORT models). Independently of the model, the main source of heterogeneity in discrimination tasks arises from protocol variations across laboratories, such as the nature of objects (size, shape, color, texture), the discriminability of the stimulus (low, moderate or high degree), the time and episodes of familiarization, sessions/inter-trial interval durations and environmental conditions (light, noise, cage size, sawdust). Additional inevitable variables like species, strain or sex add even more variability across findings and differences inside each depression models, especially in prenatal stress and maternal separation/deprivation procedures.

Studies using prenatal stress or early-life stress procedures showed either impairing [105,106,107,108,109,110,111,112,113], or improving [114,115] or no effects [105,106,108,109,110,114,116,117,118] on object recognition test. Despite a thorough analysis of all the parameters characterizing studies presented in Table 3, no common features in pre/perinatal manipulations were observed.

**Table 3:** Episodic-like memory performances in rodent models of anxiety/depression.

Behavioral Test	Animal Model	Species	Sex	Age When Tested	Interval Intertrial	Effect on Discrimination Index (DI)	Reference
WHAT?	PNS	Mouse	♂/♀	Juvenile (PN23)	4 h	No effect	[105]
			♂	Adult (PN45)		No effect	
			♀			Impairment	
		Rat	♂/♀	Juvenile (PN23)	2 h	No competent for the task	[106]
			♂	Adult (PN56)		Impairment	
			♀			No effect	
			♀	Juvenile (PN28)	1 h	No competent for the task	[107]
				Adult (PND90)	1 h	Impairment	
			♂	Adult (PND80)	1 h	Impairment	[108]
			♀		1 h	No effect	
			♂	Adult (PND60)	40 min	Improvement	[114]
			♀		40 min	No effect	
			♂/♀	Adult (PND63)	15 min	No effect	[109]
			♂		1 h	No effect	
			♀			Impairment	
			♂		3 h	Impairment	
			♀			Impairment	
			♂/♀	Adult (PND63)	24 h	Impairment	[110]
	MS	Mouse	♀	Adult (PND85)	6 h	Impairment	[111]
			♂/♀	Adult (PND60)	24 h	Impairment	[112]
		Rat	♂	Adult (PND60)	1 h, 4 h	Impairment	[113]
			♂	Adult (PND70)	1 h	No effect	[116]
			♂	Adult (PND75)	1 h	No effect	[117]
					24 h		
			♂ & ♀	Adult (PND90)	2 h	No effect	[118]
			♀	Adult (PND55)	1 h	Improvement	[115]
					24 h		
	MD		♂	Juvenile (PND35)	1 h	No effect	[119]
			♀			Impairment	
			♂/♀	Adult (PND60)	4 h	Impairment	[120]
	Early stress life	Mouse	♂/♀	Adult (PND > 90)	24 h	Impairment	[121]
	Social defeat	Mouse	♂	Adult	1 h	Impairment	[122]
					24 h	Impairment	
	UCMS	Mouse	♂		1 h, 2 h	Impairment	[123,124,125]
					24 h	Impairment	[126]
		Rat	♂		1 h	Impairment	[125,127,128]
	CORT	Mouse	♂		5 min	Impairment	[102]
					1 h	Impairment	[129,130]
					24 h	Impairment	
		Rat	♂/♀		1 h	No effect	[118]
WHERE?	PNS	Rat	♂	Adult (PND80)	1 h	Impairment	[108]
			♀		1 h	No effect	
	Early stress life	Mouse	♂	Adult (PND > 90)	24 h	Impairment	[121]
			♀		24 h	No effect	
	MS	Rat	♂	Adult (PND75)	1 h	No effect	[117]
					24 h	Improvement	
	UCMS	Mouse	♂	Adult	24 h	Impairment	[126]
		Rat	♂		4 h	Impairment	[127]
WHEN?	PNS	Rat	♂	Juvenile (PND30–40)	1 h	No effect	[131]
	MS			Adult (PND75)	1 h, 3 h	Improvement	[117]
				Adult (PND60)	3 h	Impairment	[132]

On the contrary, object recognition memory is predominantly impaired in chronic stress procedures applied during adulthood, such as social defeat, UCMS or the CORT model. This alteration is indicated by a diminution of the discrimination index (DI) [102,122,123,124,125,129,130], regardless of the duration of the inter-trial delay. One of these studies assessing long-lasting effects of UCMS procedure showed that anxio-depressed animals still displayed a discrimination deficit one month after the end of the stress treatment [125], suggesting that episodic-like alterations persist even after the cessation of the stress. These findings are in line with episodic memory deficits remaining in remitted or recurrent MDD patients [96,97]. A single study performed in rats disproves this impairment in the CORT model observing no effect in anxio-depressed animals [118]. Besides a likely species-dependent performance, the 2-week corticosterone protocol used in this study compared to a 4-week one in others studies [102,129,130] could explain in this case the lack of effect on recognition memory.

### 4.2. Where and When?

Although the novel object recognition test is widely used in laboratories, place recognition (the “Where” component) and temporal ordering (the “When” component) tasks can help to refine the degree of evaluation of episodic-like memory. The place recognition task examines the animal’s ability to discriminate between an object that was moved during the delay between the acquisition and the retention phase and an object that remained stationary, adding a spatial component to the task. On the other hand, temporal ordering task consists in presenting a set of two identical objects during a first acquisition session. After a defined interval, another set of two new identical objects is presented to animals for exploration. During the retention session, animals have to discriminate between the “old object” from the first acquisition session and the “recent” object from the 2nd session. An intact temporal order memory is observed when an animal has spent more time with the “old” object [99]. 

The same pattern emerges from place recognition memory compared to object recognition with conflicting pre/peri-natal findings (impaired: [108,121]; improved: [117]; no effect: [108,117,121]) *versus* adult chronic stress procedures (impaired: [126,127]). In this type of recognition, female animals across models seem to be less vulnerable than male animals [108,121].

Very few studies evaluated the “when?” component of episodic-like memory *via* the temporal ordering task. Only works in maternal separation were performed, once again presenting discrepancies in the results. One study showed no effect of MS procedure on temporal order memory [133], while another one found an impairment [132]. At this time, there is not enough information available in the study of place recognition and temporal ordering tasks to clarify the impact of anxiety-depression models on these episodic-related tests.

Independently of the nature of task, antenatal and early post-natal procedures were the only models in which an improvement in episodic-like performances was observed [114,115,117]. For example, Makena *et al.* [117] showed that object location and temporal ordering task performances were enhanced in maternally separated rats, suggesting that perinatal manipulations can positively modulate brain development and neuro-adaptative responses to a stimulus, influencing the animal behavior reactivity as adults. Furthermore, post-natal manipulations have been reported to increase the intensity of maternal care (during reunion after a given period of dams-pups separation) of maternally-separated pups after a brief separation [134,135], and to provide greater skills to adapt to psychological and physiological stressors in adulthood [115,136].

Taken together, except an overall impairment in all discrimination tasks observed in adult chronic stress procedures, results are too inconsistent in pre/perinatal manipulations to extract relevant advances in the area of episodic-like memory. Further investigations focusing on place and temporal recognition may contribute to progress in the discovery of fundamental mechanisms underlying episodic-like memory in rodents in a pathological context. Finally, efforts should be intended to integrate episodic memory in clinical neuropsychological tests performed in MDD patients to fully assess cognitive functions.

## 5. Working Memory Deficits in Rodent Models of Anxiety/Depression

In humans, the concept of working memory refers to the ability to get and temporarily store specific information during a variable delay in which the stimulus is absent, and to return the message when requested by the context. Clinically, the majority of neuropsychological tests assessing working memory relies on verbal tasks such as the Digit Span task (typically remembering a phone number or a list of word items and repeating them back after a defined delay) [72,75]. In rodents, working memory can be defined as a “short-term memory for an object, stimulus or location used within a testing session, but not typically between sessions” [137]. Unlike reference memory (long-term association between the stimulus-response pairing), working memory is characterized by its transience because it needs to function on a particular trial and then must be forgotten and ignored in following trials. Although using language as a parameter is not achievable in rodents, many behavior paradigms have been created to model this type of memory. The most widely used preclinical tools to assess working memory are maze type tasks which require spatial working memory to be solved. Some of the common variants of these tasks used in MDD context are T-maze and Y-maze alternation tasks, which rely on the natural exploratory behavior of rodents and exploit animals’ inherent tendency to choose an alternative arm over an arm that has been previously explored on consecutive trials [11,137]. Moreover, protocol adaptations in spatial learning tasks such as the radial arm maze (RAM) or Morris water maze (MWM) are often used to assess working memory in chronically stressed animals [137,138]. For example, whereas the spatial version of MWM evaluates reference memory (the location of the hidden platform remains the same across all the trials), the cued version of MWM enables the assessment of working memory (the location of the hidden platform is switched between each session), forcing animals to recruit different neuronal pathways and mechanisms to succeed the task.

As illustrated in Table 4, working memory performances in rodents in various anxiety-depression contexts appear to be model-dependent. Indeed, cognitive performances vary according to the nature of the chronic stress procedures. While prenatal stress and unpredictable chronic mild stress procedures both induce global impairment in working memory, maternal separation and chronic corticosterone methods seem to have no impact on this type of memory. Interestingly, impairment or absence of effect in working memory is consistent in each different preclinical model whether alternation tasks or adapted RAM or MWM were used, suggesting that working memory stress-induced effects are not task-dependent.

**Table 4:** Working memory performances in different models of anxiety/depression.

Cognitive Domain	Model	Species	Gender	Effect on Working Memory	Reference
Working alternation task: T-maze or Y-maze	PNS	Rat	♂/♀	↓ alternation	[139,140,141]
	MS	Rat	♂/♀	No effect	[118,119,142,143,144]
		Mouse		No effect	[112]
			♂	Strain-specific effects	[145]
	Social defeat	Mouse	♂	↓ alternation	[146]
		Rat	♂	Delay-specific effects	[147]
			♂/♀	No effect	[148]
	CMS	Rat	♂	↓ alternation	[149,150,151,152]
		Mouse		↓ alternation	[153]
	CORT	Rat	♂/♀	No effect	[118]
			♂	No effect	[118,142,144,154,155]
Working spatial memory: cued MWM/BM/RAW	PNS	Rat	♂/♀	No effect	[109]
				Learning impairment	[140]
	MS	Rat	♂/♀	No effect	[156]
			♀	No effect in learning	[115]
				Enhancement in retention	
	Social defeat	Rat	♂	Delay-specific effects	[157,158]
	CMS	Rat	♂	Learning impairment	[159]
				Retention impairment	[160]
	CORT	Rat	♂	No effect	[161,162]

Bidirectional findings are observed when analyzing early chronic stress life procedures effects on working memory. Indeed, while prenatal stress mostly impaired alternation tasks and spatial working memory [139,140,141], these cognitive performances are unchanged in most maternal separation studies [112,115,118,119,142,143,145,156]. Specifically, working memory following maternal separation performed in mice was found to be strain-specific. In the delayed alternation T-maze, Balb/cJ mice displayed a spatial working memory deficit whereas C57Bl/6J mice were not affected [145]. In prenatal stress, all studies describing an alteration in working memory were performed in juvenile rats (approximately 1 month of age, from PND 26 to PND 36) [139,140,141]. The only study performed in PNS-young adult rats failed to demonstrate any behavioral changes in the radial-arm maze [109], suggesting that cognitive working functioning may be more sensitive to PNS procedure in juvenile than in adult rats. In addition, Markham *et al*. provided a full characterization of cognitive consequences in PNS-rats [106] in which working memory tools were based on discrimination processes. In this study, all the learning and retention parameters of visual/spatial discrimination or visual discrimination alone were negatively impacted by chronic gestational stress, suggesting in this case as a perseverative behavior as a consequence of prenatal stress. 

Regardless of the nature of the task, a robust altered effect in unpredictable chronic mild stress animals was found on working memory. Indeed, all of the preclinical studies demonstrated either a decrease in alternation behavior [149,150,151,152,153] or a decrease in learning and retention abilities in spatial working memory after a chronic unpredictable environmental stress [159,160]. On the opposite, working memory was not affected in rats submitted to a chronic corticosterone administration [118,142,144,154,155,161,162]. This could be explained by the cessation of the chronic CORT exposure before behavioral testing, as found in a recent study examining prior effects of chronic CORT stress in the cued version of MWM [162]. Moreover, working memory may be sensitive to the duration of chronic CORT, as a longer duration of CORT regimen (56-days CORT treatment instead of a usual 21 to 28 days treatment duration) can induce a spatial working memory impairment in the Y-maze in rats [154].

Interestingly, several studies initially focused their attention on neonatal maternal separation and chronic CORT procedures independently before combining these two methods (MS + CORT) to assess their behavioral effects in delayed alternation tasks [118,142,144]. While all of these works agreed on the absence of working cognitive effects of these procedures separately, it seems that results concerning the combined MS + CORT procedures are task- and delay-dependent. Following the combined MS + CORT procedure, working memory assessment via the Y-maze demonstrated a decrease in the percentage of time spent in the novel arm after a 2 h-delay [118,142], whereas no cognitive effect in alternation rate was found in any of the tested delays (30 and 60 s) in the T-maze [144]. 

Finally, working memory performances in social defeated animals varied, in delay- and species-dependent manners. In the T-maze alternation task, mice displayed a decrease in alternation rate as soon as the 5-seconds delay [146] whereas the alternation rate was not affected in rats, neither at 10 or 30 or 60 s delays [147,148]. The delay must rise to 90 s to induce a deficit in working memory in adult defeated-rats compared to controls [147]. This observation supports that: (1) the longer the delay between sessions, the better the probability to forget the information; and (2) chronic social stressed mice are more vulnerable than defeated-rats in working memory tasks.

Taken together, chronic environmental stress procedures lead to a decline in working memory whether it was applied during gestational or adulthood periods. On the contrary, early maternal separation or chronic corticosterone regimen do not cause any damaging effects separately, but demonstrated conflicting delay-dependent effects when associated. Socially-defeated animals displayed species and delayed-dependent behaviors in working memory. Other non-spatial behavioral paradigms evaluating working memory with operant tools are currently in development such as Delayed Match to Sample, Delayed non-Match to Sample Delayed [137], but so far, no reports were found in an anxiety-depression context. 

## 6. Attention and Executive Functions in Rodent Models of Anxiety/Depression

Attention deficits are referenced as a diagnostic criterion in DSM-V [1] and are often reported in patients suffering from MDD, increasing the need to better understand mechanisms underlying this cognitive process through rodent models. Attention processes can be divided in three sub-domains including: the ability to sustain attention on a specific task over a long period of time (sustained attention), the ability to respond simultaneously to multiple tasks (divided attention) and the ability to focus on selective environmental information while ignoring distractions (selective attention) [163]. However, a small amount of studies examined attentional abilities in animal models of anxiety/depression (Table 5). In preclinical studies, one of the most widely used tasks measuring attention is the 5-choice serial reaction time task (5-CSRTT). Variations in this protocol can be arranged in order to assess the three subtypes of attention in a single behavior paradigm [163]. Briefly, this task consists in presenting five spatial different apertures to animals in an operant chamber, with a food cup located on the opposite wall of the five location holes. The animal initiates a trial by opening the food cup door. After a short delay, a visual signal consisting of a brief illumination of one of the five apertures is presented. If the animal nose pokes in the corresponding hole, a food pellet is delivered into the food cup and this action is counted as a correct response to the test. Conducted through several trials, attention performance is usually measured by examining when the animal responds to a correct or incorrect hole where the light appears (accuracy *versus* omissions) and the speed with which the animal responds (time reaction). Complexity in the task can be increased by varying the stimulus light or inter-trial interval duration for example, assessing in this case parameters such as premature or perseverative responses (nose poke that occurs prior to light presentation or repeated nose pokes in a previously lit aperture), giving further information on impulsive behavior (see [164,165,166] for details in methodology). Among few available reports presented in Table 5, all of them agreed about some deleterious effects of anxiety-depression on attention performances in the 5-CSRTT [167,168,169,170], increasing accordingly as cognitive challenges in the task are getting more complex. For example, under standard 5-CSRTT conditions (fixed inter-trial interval (ITI) and stimulus durations (SD)), there were few differences in performances between rats submitted to prenatal stress procedure and controls. However, when ITI and SD vary across trials, there was a decrease in accuracy and an increase in number of premature and timeout responses, respectively, indicating an impairment in sustained attention, increased impulsivity and cognitive inflexibility in prenatal stressed rats [168,171]. 

Accumulating evidence suggests that unrestrained secretion of corticotropin-releasing hormone (CRH) can contribute to the apparition of depressive symptoms as well as cognitive deficits ([172] for review). Interestingly, attentional abilities have been evaluated with the 5-CSRTT in a genetic mouse model based on overexpression of CRH throughout development. Disrupted attention processes were observed once the task was acquired, as CRH transgenic mice showed a decrease in the percentage of correct responses, a longer correct response latency and an increase in omitted responses compared to wild-type animals [167]. In line with this work, others studies using pharmacological cerebral CRH administration in mice demonstrated attention alterations [173,174].

**Table 5:** Attention and executive functions performances in different rodent models of anxiety/depression.

Executive Function	Task	Model	Species	Gender	Behavioral Effect	Reference
Attention/Impulsivity	5-CSRTT	CRH-KO	Mouse	♂	Impairment	[167]
		PNS	Rat	♂/♀	Impairment	[168]
		CMS		♂	Impairment	[169]
		CORT		♂	Bidirectional effects	[170]
Attentional set-shifting task	ASST	MS	Mouse	♂/♀	Strain-specific impairment	[145]
			Rat	♂	Impairment	[132]
		UCMS			Impairment	[175,176]
		CRS			Impairment	[177]
		CORT			Impairment	[178]
Reversal learning	MWM	MS	Mouse	♂	Reversal learning impairment	[111]
				♀	No effect	
			Rat	♂/♀	Reversal learning impairment	[179]
					Reversal learning enhancement	[180]
		Social defeat	Mouse	♂	Reversal learning impairment	[181]
				♀	No effect	
		UCMS	Mouse	♂	Reversal learning Impairment	[182]
			Rat	♂	Reversal learning impairment	[183,184]
		CORT	Mouse	♂	Reversal learning/retention impairment	[102]
			Rat	♂	Reversal learning impairment	[161]

A recent study further investigated impulsive behavior in adolescent CORT-treated rats in the 5-CSRTT and others attentional/impulsive tasks derived from multiple-choice serial reaction tasks [170]. Intriguingly, adolescent chronic exposure to corticosterone induced in bidirectional effects on impulsivity behavior. CORT-treated rats were slightly less impulsive on measures of impulsive action, but also markedly displayed an increased impulsive choice, selecting the immediate small reward faster and more frequently instead of waiting for the larger reward after a longer delay. This suggests that chronic CORT treatment in adolescent rats enhances impulsive choice while simultaneously decreases impulsive action [170]. Collectively, these findings showed that an anxio-depressed state in rodents can alter attentional processes across models.

Executive functions refer to higher cognitive processes in mental functioning such as: cognitive flexibility, planning, decision-making, reasoning, multi-tasking, concept formation or response inhibition and represent by far the most complex and challenging domain to model cognitive deficits in rodents. Similarly to attentional deficits, executive dysfunctions are commonly listed as cognitive difficulties in MDD patients and are progressively pointed out as specific neurocognitive abnormalities observed in remitted, recurrent or melancholic subtype of depression. Table 5 gathers available literature about executive functions performances across different anxiety-depression rodent models. 

Among all the aspects of executive functioning, cognitive flexibility remains the most widely used task and can be modeled in preclinical studies in different ways. Particularly, the most described task used to assess cognitive flexibility in rodents in anxiety-depression models is reversal learning in spatial learning and memory tests. Classically, animals learn, during an acquisition phase, the specific location of an escape platform, which is then switched to the opposite location during the reversal phase, asking them to adapt and change their behavior to succeed in the task. Overall, the majority of studies showed impairments in cognitive flexibility in reversal learning and memory [102,111,161,179,181,182,183,184], evoked by the incapacity for stressed animals to decrease the time to find the platform along reversal trials and to spend more time in the new target quadrant instead of the previous target quadrant during the retention trial. These findings suggest that anxio-depressed animals lost their ability to unlearn a previously acquired rule and to adapt their behavior to the new instruction, in accordance with clinical observations.

Interestingly, some of these studies reported that chronic stress procedures, independently from their nature, induced deficits in spatial learning reversal without affecting acquisition learning [161,182,183,184]. It is assumed that chronic stress can induce a perseverative behavior in MWM, preventing animals from re-learning a new location. This possibility was supported by the measurement of a decrease in escape latency during acquisition (confirming that a correct learning occurs) followed by an increase in the percent of time spent in the initial training quadrant during reversal learning [184]. Other authors suggest that dissociation between acquisition and reversal cognitive performances could be due to the involvement of specific brain regions. Indeed, whereas the hippocampus is primarily involved in spatial learning tasks, the prefrontal cortex, implicated in reversal learning, reveals a greater vulnerability to the deleterious effects of chronic environmental stress [182,183,185].

Sex-specific differences have been observed in studies performed in mice, showing that female animals were resilient to cognitive alterations effects observed in male animals [111,181]. While some expected elements, based on sexual differences such as gonadal hormones modulation, could explain the male vulnerability to cognitive effects of stress, other causes directly inherent to the nature of the chronic stress procedure or the cognitive task itself might are open to discussion. For example, supporting the brain region-specific hypothesis previously evoked, spatial acquisition was unaffected in socially defeated mice in both sexes, but only males failed to succeed in the reversal learning phase, suggesting that neuronal circuits involved in cognitive flexibility are differently impacted in male and female mice by chronic social stress [181]. On the other hand, in a chronic early-life stress context, differences in dams’ interaction towards male or female offspring [186] may explain why female offspring are less vulnerable to the effects of MS than males [111]. Further investigations are required to identify mechanisms underlying sex-specific effects of anxiety-depression context on cognitive reversal performances.

As well as episodic-like memory, maternal separation was the only model in which an enhancement of cognitive flexibility was found. In the only study showing improved reversal learning effects, authors tested cognitive performances in maternally-separated rats at three different ages of development: PND21 (after weaning), PND35 (juvenile) and PND56 (young adult). Their results highlighted that enhanced behavioral effects generated by MS procedure do not emerge until adolescence [180].

The attentional set-shifting task (ASST) is a paradigm used to assess cognitive flexibility in anxiety-depression models. Probably less employed than reversal tests because of its complexity to set up, this task refers to animal’s ability to recognize and to adapt their behavior to various changing rules or environmental circumstances. In the most common version of rodent ASST, rodents are trained to dig for a food reward and make a simple discrimination based on both odor and digging medium. During the test, at each stage, rats are required to attend to one of two perceptual dimensions (odor or digging medium), and to shift between stimuli dimensions in order to learn the rule to successfully retrieve the food reward. The ability to extract knowledge from different stimuli dimensions suggests that animals are capable of using some aspects of higher-order cognitive functions present in human executive functioning [187,188].

Although the nature of parameters varies according to the study (discrimination, compound discrimination, reversal or extra-dimensional phases), an alteration in cognitive flexibility has been observed across anxiety-depression models [132,145,175,178]. A study performed in mice revealed a strain-specific impairment, where an increase in days to reach the criterion in the extra-dimensional stage was observed in maternally-separated Balb/c but not in C57Bl/6 mice [145]. This strain-specific performance could be explained by differences in maternal care (more nursing, licking and grooming in C57Bl/6 mice than Balb/c) and genetic susceptibility to stress.

Studies performed in rats highlighted interesting differences in performance between chronic stress procedures. The first work studying the consequences of MS on cognitive flexibility showed that maternally-separated rats displayed weaker overall performances in the ASST compared to control animals, as indicated by significant deficits in compound discrimination (SD), reversal 1 (REV1) and extra-dimensional set-shifting (ED) [132]. These three stages are characterized by the appearance of a new level of complexity never encountered before by the rat, illustrating their reduced ability to adapt to the introduction of new complex rules and perform strategy changes. Animals exposed to a chronic environmental stress procedure during adulthood (UCMS or chronic restraint stress) displayed a constant deficit in extra-dimensional shift (ED) in ASST [175,176,177]. To evaluate the persistence of this cognitive deficit, the performance of separate groups of rats was assessed on the 4th, 7th, 14th and 21st day following the cessation of chronic restraint stress [177]. Stressed rats exhibited long-lasting ED set-shifting impairments, since these deficits were observed at all the time points, even three weeks following stress termination. Finally, CORT-treated rats were less efficient in all the reversal learning stages of the ASST (REV1, REV2, and REV3) than vehicle-treated animals, due to a failure to learn the new rule or to unlearn the old rule. Moreover, CORT-treated rats required more trials at the intradimensional shift stage (ID) suggesting that they treated each set of cues independently and did not use their prior experience as an indicator of which dimension was the salient one [178]. 

Despite the complexity of assessment instruments and interpretation, cognitive deficits in attention and executive functions are shared across all anxiety-depression existing rodent models, strengthening clinical findings in MDD patients. Developing further preclinical behavioral tools to explore other aspects of executive functions than cognitive flexibility is obviously the next challenge in the field.

## 7. Spatial Learning and Memory Deficits in Rodent Models of Anxiety/Depression

Spatial memory is one of the most documented types of memory in laboratories, thanks to the development of various spatial behavioral paradigms through decades (i.e. Morris water maze, 8-radial arm (water) maze, Barnes maze, Y-maze, novel object recognition location). This particular form of reference memory has already been the target of a bibliographic review focusing on the consequences to chronic stress effects on spatial learning and memory performances, but mostly limited to chronic restraint stress or social exposures in several species [189]. To our knowledge, no synthesis gathering data about spatial memory performances in anxiety-depression rodent models was constituted. One of the main interests of studying spatial memory in rodents lies into the intimate relationship between hippocampus, chronic stress and spatial skills, allowing investigators to dissect mechanisms underlying spatial learning and memory processes *via* rodent models. It is now well known that long-term exposure to a chronic stress can induce deleterious changes in hippocampal structure, including in the neurogenesis process, thus deteriorating some aspects of spatial behavioral experience [121,190].

Historically, the three main mazes, mentioned in Table 6 used in preclinical studies in anxiety-depression models, were created between the late 1970s and early 1980s. The Morris water maze (MWM), certainly the most widely used spatial memory paradigms, relies on distal extra cues surrounding the pool to guide animals to navigate from start locations to locate a hidden escape platform [138,191]. The Barnes maze (BM), a dry-land version of the MWM, requests animals to escape from a brightly lit exposed circular open platform surface to a small dark recessed chamber located under one of the 20 holes around the perimeter of the platform [192]. Finally, the radial arm maze (RAM) consists in eight equidistantly spaced arms radiating from a circular central platform. Reference spatial memory is assessed when the rodent only traverses goal arms of the maze containing a food reward [193]. Common features in spatial tasks rely on a spatial learning phase (acquisition phase) and a reference memory probe (retention phase). Spatial learning is usually assessed across repeated trials along several days of training and reference memory is determined by preference for the platform quadrant/hole when these ones are removed. These spatial tests distinguish from each other by the nature of the motivation to perform the requested task: either aversively motivated task (water in MWM, RAWM or high open arena in BM), or appetitive motivated tasks with reward (RAM) [189,191].

**Table 6:** Visuo-spatial learning and retention performances in different rodent models of anxiety/depression.

Model	Test	Species	Gender	Age When Tested	Learning	Retention	Reference
PNS	MWM	Rat	♂	Juvenile	Impairment	Impairment	[141]
			♀		No effect	No effect	
			♂/♀	Adult	Impairment	Impairment	[106,194,195]
			♂		No effect	Impairment	[195]
			♂/♀		No effect	Impairment	[196]
					No effect	Improvement	[109]
					No effect	NA	[197]
		Mouse	♂/♀		No effect	Impairment	[198]
	BM	Mouse	♂		No effect	No effect ^a^	[199]
						Improvement ^a^	
MS	MWM	Rat	♂/♀	Juvenile	No effect	No effect	[180]
				Adult	Impairment	Impairment	
					No effect	No effect	[200]
			♂		No effect	No effect	[132,179]
					No effect	Impairment	[201,202]
					No effect	Improvement	[203]
			♀		No effect	Improvement	[115]
Chronic early life stress	MWM	Mouse	♂	Adult	Impairment	Impairment	[121]
			♀		No effect	No effect	
Social defeat	MWM	Mouse	♂	Adult	No effect	No effect	[122,146]
	BM	Mouse	♂		No effect	NA	[181]
			♀		No effect	NA	
	RAWM	Rat	♂		NA	Impairment	[157]
Learned helplessness	MWM	Mouse	♂	Adult	Impairment	Impairment	[190]
UCMS	MWM	Mouse	♂	Adult	Impairment	Impairment	[123,182,190,204]
		Rat			Impairment	Impairment	[128,159,205]
CUR	RAWM	Rat	♂	Adult	No effect	Impairment ^b^	[206]
					No effect	Improvement ^b^	
					Impairment	No effect ^b^	[207]
			♀		No effect	No effect ^b^	
CORT	BM	Mouse	♂	Adult	Impairment	Impairment	[102]
	MWM	Mouse			Impairment	Impairment	
		Rat			Impairment	Impairment	[208]
	RAM	Rat			Impairment	Impairment	[209]
	BM	Rat			Impairment	Impairment	[154,160,209]
					No effect	Improvement	[162]

^a^: depending on the presence of a recovery period between chronic stress cessation and behavioral testing; ^b^: depending on the presence of a recovery period between chronic stress cessation and behavioral testing.

The vast majority of studies uses latency to reach the platform in MWM and BM tasks as a parameter to measure learning across training days. However, latency is sensitive to motor and motivational factors that are likely to be modified by chronic stress procedures, leading to possible critical issues for the interpretation of data. Consequently, it is essential to control distance and speed performances, to confirm that the observed reduction in latencies across trials or days is correlated to a spatial learning alteration and not to motor, exploratory or motivational factors related to an anxiety-depression phenotype. Table 6 summarizes spatial learning and memory performances in different anxiety-depression models. A first clear dissociation emerges in learning abilities across models depending on the period during which chronic stress models were applied. Most spatial studies report that chronic stress procedures performed in early-life (PNS, MS) do not compromise spatial acquisition abilities [109,115,121,132,141,179,180,195,196,197,198,199,200,201,203], whereas other adulthood anxiety-depressive phenotype inductions (LH, UCMS, CORT) result in an impairment of spatial learning [102,123,128,154,159,160,182,190,204,205,207,208,209]. 

Two hypotheses can be proposed concerning the lack of spatial learning detrimental effects following early-life stress. It is likely that specific spatial learning neuronal networks and pathways may not be influenced by these chronic stress procedures at this young age, inducing no damaging effects in adulthood either. However, spatial learning performances being tested at the adult age, brain maturation occurring during adolescent and young adult periods, during which stress procedures are no longer applied, could be sufficient to compensate cognitive learning dysfunctions that might have been induced during previous prenatal or perinatal period. 

It is also remarkable that impairment in spatial learning phase after adulthood anxiety-depression procedures is almost systematically associated by impairment in retention performances, suggesting that all the spatial abilities were impacted here [102,123,128,154,159,160,182,190,204,205,208,209]. Increasing evidence has showed that stress-induced depressive and cognitive phenotypes in adulthood are associated with a reduction of hippocampal neuronal plasticity and neurogenesis, supporting harmful effects of UCMS and CORT methods in learning and memory performances [15,210].

Among studies that assessed spatial retention memory, consequences of anxiety-depression phenotypes are more ambiguous, so that categorization according to the type and the nature of stress models appears inconceivable. According to the Table 6, chronic stress procedures modeling MDD in rodents:

- Impaired probe trial performances in twenty-two studies [102,106,121,123,128,141,154,157,159,160,182,190,194,195,196,198,201,204,205,208,209,211],
- Had no effect in nine studies [121,122,132,141,146,179,199,200,211],
- Enhanced retention performances in five studies [109,115,162,199,203].

Overall, almost 2/3 of cited studies displayed impairment in spatial reference memory, highlighting the vulnerability of retention ability in a low mood pathological context, independently from the anxiety-depression model. In early-stress life procedures (PNS and MS), factors that contribute to the high variability in results are multiple and rather easy to identify. For example, in PNS studies, the period and duration of stress procedure can affect the behavioural performance. One study examined the effects of three separate prenatal stress periods on spatial learning and memory retention in male rats: (1) before pregnancy stress during 10 days, (2) early pregnancy stress (Gestational day 0 (GD) to GD10) and (3) late pregnancy stress (GD11-GD21) [195]. Preconception and early pregnancy stress manipulations strongly impaired learning and memory performances, whereas late pregnancy stress only altered retention memory in adult offsprings, suggesting that the intensity of detrimental effects are linked to the time point of stress application. The hypothesis that the nature of stressors (physical or psychological stressors) might induce different responses on spatial performances in PNS-animals has also been recently tested [196]. Obviously, many other factors, including the nature of the task (MWM/BM/RAWM), the age of animals at the time of the test, species-, strains- and sex-specificity, contribute to the difficulty to find a distinct profile in cognitive spatial deficits in early-life stress protocols.

Interestingly, some of the studies in which spatial learning was not affected in anxio-depressed animals revealed impairments in retention trial [195,196,198,201]. Even though retention performances are intimately linked to learning processes, two distinct functioning circuits involving different brain structures and neuronal pathways may exist [212,213]. In these particular cases, it appears that learning process is more resistant to pre/perinatal chronic procedures than retention functioning. 

Studies using chronic unpredictable restraint (CUR) stress provide other pieces of information. A recent study investigated whether a recovery period between chronic stress application and spatial tests may impact behavioral responses. For that purpose, one group of rats received behavioral testing immediately after a 21 days-CUR procedure, whereas another group of animals was given 21 days to recover from chronic stress before assessing spatial performances. Immediately-stressed rats displayed a learning spatial impairment, while there was no impact on spatial learning in rats that benefited from a recovery period [207]. Focusing on retention parameters, the same pattern of results with an improvement in spatial reference memory after a recovery period was found in rats [206], confirming the reversibility of CUR-induced deficits in this hippocampal-dependent spatial learning and reference memory task.

Interestingly, administration of CUR in female rodents failed to alter spatial learning and memory performances, whether they had a recovery period or not. This comment is in line with all the other studies that have been conducted in female mouse or rat, because they did not report any changes in spatial learning and retention behaviors following different chronic stress procedures [115,121,141,207]. The female resilience to cognitive spatial alterations observed in males in an anxiety-depression context is not an isolated phenomenon and was also apparent in other types of memory such as episodic-like memory [106,108,121] and reversal learning tasks [111,181]. Further studies are required to clearly differentiate sex-specificities of cognitive performances in a MDD context.

## 8. Associative Memory in Rodent Models of Anxiety/Depression

Up to now, the vast majority of clinical studies using neuropsychological tests in MDD focused on cognitive deficits such as executive functions, attention, processing speed, working memory, verbal and visual learning and memory or psychomotor performances. Clinical emotional assessment remains paradoxically one of the less documented type of memory studied in MDD. Only few reports concentrate their research specifically on cognitive biases associated with mood disorders [73]. However, anhedonia, one of the core symptoms in the DSM-V, is defined as the loss of interest in originally rewarding and enjoyable activities. Precisely, cognitive biases in depressed patients refer to dysfunctions in emotional memory including distorted information processing and cognitive bias favoring sad information and lower responsiveness to positive outcomes [20,214,215].

Emotional memory (associative memory in rodents) refers to a non-declarative type of memory, implicit memory, characterized by the impossibility to reach the memory through a conscious process. Emotional memory is necessary for individuals to learn to predict a danger and adapt their behavior accordingly. In their natural environment, rodents respond to danger in a species-specific manner by a typical freezing behavior triggered to avoid detection from possible predators. Freezing is defined as a cessation of all movements, except breathing. This instinctive behavioral response is used in preclinical studies to assess associative learning and memory. In anxiety-depression models, this type of memory is evaluated using classical conditioning tasks (contextual or cued fear conditioning; passive avoidance test), based on negative reinforcers, and being dependent on the hippocampal brain structure [216,217]. Basically, animals learn to associate a conditioned stimulus (context, light, tone) with an aversive and inescapable unconditioned stimulus (mild foot shock applied through the grid floor) during an acquisition session. During the test session, animals are re-introduced in the conditioned chamber and are submitted to either a cued fear conditioning (tone or light presentation in a modified context) or a contextual fear conditioning (similar context without footshock) [91]. This situation theoretically increases the freezing behavior in animals with an intact associative memory. 

Findings presented in Table 7 about fear conditioning and fear extinction behaviors in anxiety-depression models are conflicting. We separated pre/perinatal models from adulthood-induced models. Early-life chronic stress and perinatal manipulations induced either fear conditioning impairments [106,111,112,218] or no effects on associative memory [105,219,220], whereas chronic stress procedures performed in adulthood (social defeat, learned helplessness, unpredictable chronic mild stress or chronic corticosterone administration) improved in associative memory in comparison to control animals [146,149,221,222,223,224,225,226,227]. These latter results may actually be in line with the observation that patients suffering from MDD display a better memory for negative stimuli than neutral or positive ones.

**Table 7:** Associative memory performances through conditioning tasks in different models of anxiety/depression.

Type of Task	Model	Species	Age When Tested	Sex	Fear Conditioning	Fear Extinction	Reference
Contextual/cued associative task	PNS	Mouse	Weaning	♂/♀	No effect	No effect	[105]
			Juvenile		No effect	No effect	
		Rat	Adult		-	Impairment	[208]
					Impairment	No effect	[106]
	MS	Mouse	Adult	♂/♀	Impairment	-	[111,112]
		Rat	Juvenile		Impairment	No effect	[218]
			Adult		Impairment	Impairment	
				♀	No effect	No effect	[219]
					No effect	-	[220]
	Social defeat	Mouse	Adult	♂	Improvement	-	[146,221,227]
	Learned Helplessness	Rat			Improvement	Impairment	[222]
	UCMS	Rat			Improvement	-	[149,223]
	CORT	Mouse			Impairment	-	[102]
		Rat			No effect	Impairment	[228]
					No effect	No effect	[155]
					Improvement	-	[224,225,226]

A recent study published in *Nature* showed that the activation of positive memory could reverse depression-like behavior. Optogenetic reactivation of dentate gyrus cells previously active during a positive experience can rescue stress-induced depression-related behaviors [229]. This work identified glutamatergic activity in the hippocampus-amygdala-nucleus-accumbens pathway as a candidate circuit supporting positive memory. Studies revealing a decrease in freezing duration compared to controls are also in accordance with an associative memory alteration since animals are unable to associate and distinguish an unsafe context from a safe one [230]. Here, opposite effects in the same task can be associated with behavioral associative impairment: an increase in freezing behavior may be interpreted as an enhancement in memory for negative stimuli, while a decrease in freezing behavior may be translated as a difficulty to associate a specific context with an aversive stimulus. 

When the conditioned stimulus is repeatedly presented without the presence of the unconditioned stimulus, it corresponds to the extinction phase. Consequently, freezing behavior decreases over time because the context is no longer associated with the danger. In anxiety-depression models, assessment of extinction fear remains less investigated than fear conditioning. Among studies evaluating this aspect of associative memory, no consistent findings were described, suggesting in stressed-animals either conserved fear extinction performances [105,106,155,218,219] or the incapacity to adjust their behavioral response to a neutral stimulus [218,222,228]. Deficits in fear extinction are rather often reported in post-traumatic stress disorders (PTSD) models in rodents, consistent with the incapacity of PTSD patients to distinguish the real traumatic situation they lived in the past from a neutral context situation in which an unconditioned stimulus related to the traumatic memory has been inserted. Although PTSD confers specific significant psychiatric disturbances and functional impairments, depression and PTSD are commonly co-morbid diseases. Research suggests that significant depressive symptomatology affects 30% to 50% of subjects diagnosed with PTSD [231]. Impairment in fear extinction could also be interpreted as a perseverative behavior [232]. Further studies are needed to identify the distinct role of fear extinction in MDD context.

The step-through passive avoidance test is a hippocampus-dependent memory task conducted in two sessions. The apparatus is divided in two chambers separated with a guillotine door: one illuminated, transparent “safe” compartment and one darkened, opaque “shock” compartment. During the first testing day, animals are put into the lighted compartment, in which they are expected to enter freely into the dark compartment. Once the animal has entered into the dark compartment, one inescapable foot shock is delivered. 24 h later, the animals are placed in the light compartment again for a retention test, with free access to the dark compartment without any shock. The latency for the subject to enter into the dark chamber is recorded [233]. The passive avoidance task offers a compelling complementarity to classical conditioned tasks previously presented. Instead of “simply” recognizing the environment in which they were previously shocked, animals need to inhibit their natural tendency to enter the dark chamber. This particular demand in avoidance behavior indicates the different neuronal pathways that regulate behavioral responses in this test compared to classical conditioned tasks. Involvement between the amygdala and other areas of the prefrontal cortex that mediate behavioral control are requested in avoidance mechanisms, whereas associative learning tasks is mediated by a hippocampus-amygdala circuitry [223].

Almost all the preclinical studies in the passive avoidance test found a significant decrease in latency to enter into the dark compartment in chronically-stressed animals compared to controls (Table 8), highlighting an overall deficit in associative memory conserved across anxiety-depression models [116,139,140,196,197,208,223,233,234,235,236,237]. This confirms, once again, the difficulty for depressed animals to associate the aversive stimuli with the context they met the day before and their incapacity to restrain their instinct. Similarly to classical conditioned tasks, passive avoidance measures can lead to a double-interpretation. According to studies, lower latencies to enter into the dark chamber can be interpreted as either a failure to associate the context with the aversive stimulus and the incapacity to inhibit their natural tendency to escape in a safer place, or an increase in an impulsive behavior [223].

**Table 8:** Associative memory performances in passive avoidance task in different rodent models of anxiety/depression.

Type of Task	Model	Species	Gender	Latency to Enter into the Dark Compartment Compared to Controls	Reference
Passive avoidance task	PNS	Rat	♂	Decreased	[139,197]
			♂/♀	Decreased	[140,196,233,234]
	MS	Rat	♂	Decreased	[116,235]
	Social defeat	Mouse	♂	No effect	[238]
	CMS	Rat	♂	Decreased	[223,236]
				No effect	[149]
	CORT		♂	Decreased	[208,237]

To conclude, despite some conflicting results across chronic stress models, preclinical studies appear to replicate some of the associative deficits observed in MDD patients, underlining a propensity for stressed-animals to remember negative stimuli and difficulties to associate aversive events to a specific context. Moreover, alterations in fear extinction remind symptoms observed in PTSD patients. Further efforts are needed to identify measurable relevant parameters in associative memory behavioral paradigms that can lead to a greater interpretation.

## 9. Cognitive and Emotional Deficits in Rodent Models of Anxiety/Depression and Their Relationship with Hippocampus Function

### 9.1. Hippocampal Formation and Its Role in Cognitive/Emotional Deficits

Interestingly, some recent studies revealed that dorsal and ventral poles of the dentate gyrus of the hippocampus were functionally distinct [239,240]. A lesion study supported that the dorsal hippocampus was involved in learning and spatial memory, whereas the ventral hippocampal may regulate emotional and motivated behaviors [239]. Specifically, by using an optogenetic approach, another recent study demonstrated that dorsal granule cells might contribute to spatial and contextual learning (not memory retrieval), while ventral DG cells exert a major effect on anxiety-like behavior [240].

In humans, magnetic resonance imaging (MRI) studies revealed that first-episode MDD patients had smaller hippocampi than healthy subjects and these data correlated to neurocognitive alterations [53]. In addition, hippocampal volume reductions were associated to persistent multiple cognitive impairments at six months in MDD patients [241]. Moreover, MDD patients showed impaired spatial memory during a navigation task [242,243], a task previously shown to reflect hippocampal activation and particularly the neurogenesis process. Further investigations using specialized neuroimaging methods and studies are needed to better understand the crucial role of adult hippocampal neurogenesis in cognitive deficits of MDD context.

### 9.2. Impact of Neurogenesis on Cognitive and Emotional Function

Adult neurogenesis occurs in two regions of the mammalian brain: the subgranular zone of the dentate gyrus (DG) and the subventricular zone (SVZ). It is now well established that adult neurogenesis process is intimately involved in mood and cognition regulations as well as in antidepressant drug response. Although adult hippocampal neurogenesis was shown to be necessary for antidepressant action in chronic-stress animal models [15,244], altered neurogenesis integrity is not an etiological factor in the apparition of MDD pathology. On the contrary, reduction of adult neurogenesis in rodents can directly affect learning and memory functions. Many recent reviews focused on the role of adult neurogenesis in cognition [245] and particularly in hippocampus-dependent memory formation [246,247,248,249]. Despite the availability of a large amount of data, findings about the involvement of neurogenesis in learning and memory processes remain contradictory. For example, some studies demonstrated that adult hippocampal neurogenesis process was required in rodents to perform contextual fear memory [216,217,250,251], fear memory extinction [252] or spatial learning and memory tasks [253,254,255,256]. However, other evidences do not support this role of adult neurogenesis in contextual fear conditioning [252,253,257], spatial navigation tasks [216,258,259,260] or working memory [250]. One possibility to explain such conflicting results could rely on differences in experimental designs impacting the level of difficulty of the training tasks. In other words, adult neurogenesis appears to be specifically required in more challenging and complex learning and memory tasks, such as in the pattern separation process [257,261,262]. Likewise, a study demonstrated that increasing adult hippocampal neurogenesis by enhancing survival of adult-born cells in the DG was sufficient to improve pattern separation performances in naïve mice without displaying any anxiolytic or antidepressant-like effects [263]. However, increasing neurogenesis by deleting the pro-apoptotic gene Bax from neural stem cells and their progeny was able to rescue the higher emotionality in CORT-treated mice [264].

More specifically, various approaches have allowed studying the role of the DG-CA3 circuit in pattern separation. Recently, a study showed that in animal models where neurogenesis is altered (social defeat model) or ablated (x-irradiation), a decrease in reactivation of CA3 but not of DG neurons priory activated by fear exposure was observed in a time dependent manner [265]. These results are consistent with the proposal that adult-born dentate granule cells are responsible for reactivation of memory traces in CA3, and how mal-adaptive neurogenesis state may participate in fear-generalization and stress response [266]. Overall, these preclinical observation correlates with the impaired pattern separation observed in MDD subjects [267]. Hippocampal neurogenesis inducement in adulthood, by modifying DG-CA3 circuits, may also participate in forgetting and memory clearance, depending on the strength of the initial memory [268], potentially participating to the clinical remission of MDD subjects.

The presence of cognitive alterations in mood disorders, including MDD, strongly suggests that brain structures implicated in emotional and cognitive functions are linked. However, questions of how cognitive and depressive symptoms are related to each other and how to distinguish specific biological mechanisms attributable to cognitive or emotional functions in a MDD context remain opened. Moreover, the fact that most of antidepressant drug treatments failed to treat the cognitive dysfunctions observed in MDD, despite the remission of mood-related symptoms, confirms that independent neural circuitry underlying cognitive functions and depressive symptoms may be engaged. 

## 10. Conclusions

It is now well accepted that patients with depression suffer from associated cognitive dysfunctions, such as negative or distorted thinking, difficulty concentrating, indecisiveness, reduced reaction time or memory loss. However, whether these cognitive deficits are specific to a clinical subtype of MDD in Humans (melancholic or atypical) or to a specific chronic stress procedure in animal models of the disease (pre/peri natal, adulthood or social chronic stress methods) remains to be further determined. The use of translational anxiety/depression animal models will substantially improve our understanding about these cognitive alterations.

In this review, we provided an overview of clinical findings regarding the nature of cognitive dysfunctions in depressed patients from the first episode to recurrence according to the subtype of MDD. Then, we have summarized available data indicating the type of cognitive functions (including attention, executive functions, working memory, spatial memory, episodic-like memory and associative memory) affected in different anxiety/depression animal models.

Clinical evidence indicates an overall cognitive decline in MDD patients persisting even after remission and during recurrent episodes. Among MDD subtypes, melancholic patients were identified to experience more pronounced cognitive deficits, suggesting a different pattern of cognitive impairment from those found in non-melancholic patients. Among all the cognitive alterations, executive and attentional functions deficits seem to be a constant hallmark in MDD patients, since it is present in all stages of the MDD course and in melancholic patients. Through the study of different anxiety/depression animal models, it appears that these executive and attentional deficits are conserved in rodents regardless of the chronic stress procedure. About the other types of memory, results are more heterogeneous according to the chronic stress methods used. This review points out the limit of rodent models, since heterogeneity in the results was observable within models and/or within a same type of memory. Numerous factors such as sex, protocol details, species, strain, environmental conditions, and the nature of the test promote this variability. Besides, animal models cannot fully replicate all of the cognitive deficits observed in human MDD, but rather only reflects different etiologies or aspects of the disease. Overall, among all the suggested models, adulthood-induced chronic stress models (the UCMS and the CORT model for example) seems to show greater similarities with the cognitive symptoms observed in MDD patients, key advantages compared to pre or perinatal chronic stress procedures. 

Unfortunately, cognitive condition in MDD patients tends to receive less attention than other depressive-related symptoms, particularly when it comes to consider the therapeutic strategy. Indeed, finding therapeutic strategies that treat both depressive and cognitive-related symptoms remains one of the main challenges in this field. Up to now, clinical reports about the efficacy of classical antidepressant drugs are relatively inconsistent [46,47,50,51,185]. Recent preclinical results are in line with clinical disparate findings using antidepressant drug therapies, specifically with selective serotonin reuptake inhibitors (see [192] for review). Further investigations, including non-antidepressant and innovative drugs strategies, are needed to determine which treatment could benefit to depression and comorbidities-associated signs.
